# Smart technologies and the future of concussion prevention in ice hockey

**DOI:** 10.3389/fspor.2025.1646119

**Published:** 2025-11-12

**Authors:** Arto Gråstén

**Affiliations:** Physical Education Department, College of Education, United Arab Emirates University, Al Ain, Abu Dhabi, United Arab Emirates

**Keywords:** sports-related injury, head trauma prevention, protective equipment, athlete, health

## Abstract

**Background:**

Concussions in ice hockey remain a major concern, especially among young players. Current helmets mainly protect against skull fractures but offer limited shielding from brain acceleration forces.

**Methods:**

A narrative review of epidemiological studies, helmet safety research, and technological innovations in other sports like cycling, equestrian, and football was conducted to assess limitations of current helmets and the potential for advanced protective systems.

**Results:**

Concussion rates remain relatively high, with inconsistent diagnoses making comparisons difficult. Standard helmets show minimal performance differences and have limited ability to reduce rotational forces. Innovations like wearable sensors integrated into helmets offer notable protection benefits in other sports. However, hockey-specific concerns include repeated impacts, usability, and weight limits.

**Discussion:**

Smart technologies such as airbag helmets present a viable path toward enhanced protection in ice hockey. Translating these solutions requires engineering adaptation, interdisciplinary collaboration, and cultural acceptance, especially urgent in youth sport, where long-term risks are greatest.

## Introduction

Concussion in ice hockey is an increasing concern across youth, elite, and international levels, with evidence showing rising incidence rates, longer recovery times, and lasting health effects (1–3). By raising awareness through education and modifying the hockey rink, such as introducing safety glasses, the environment can be made safer. However, these changes must be implemented carefully, as going too far could turn the sport from a contact game into a non-contact one. This paper discusses the potential of innovative technologies to prevent concussions in ice hockey, arguing that solutions like smart helmets are only a matter of time before they become viable.

## Concussions in ice hockey

**Figure 1:**
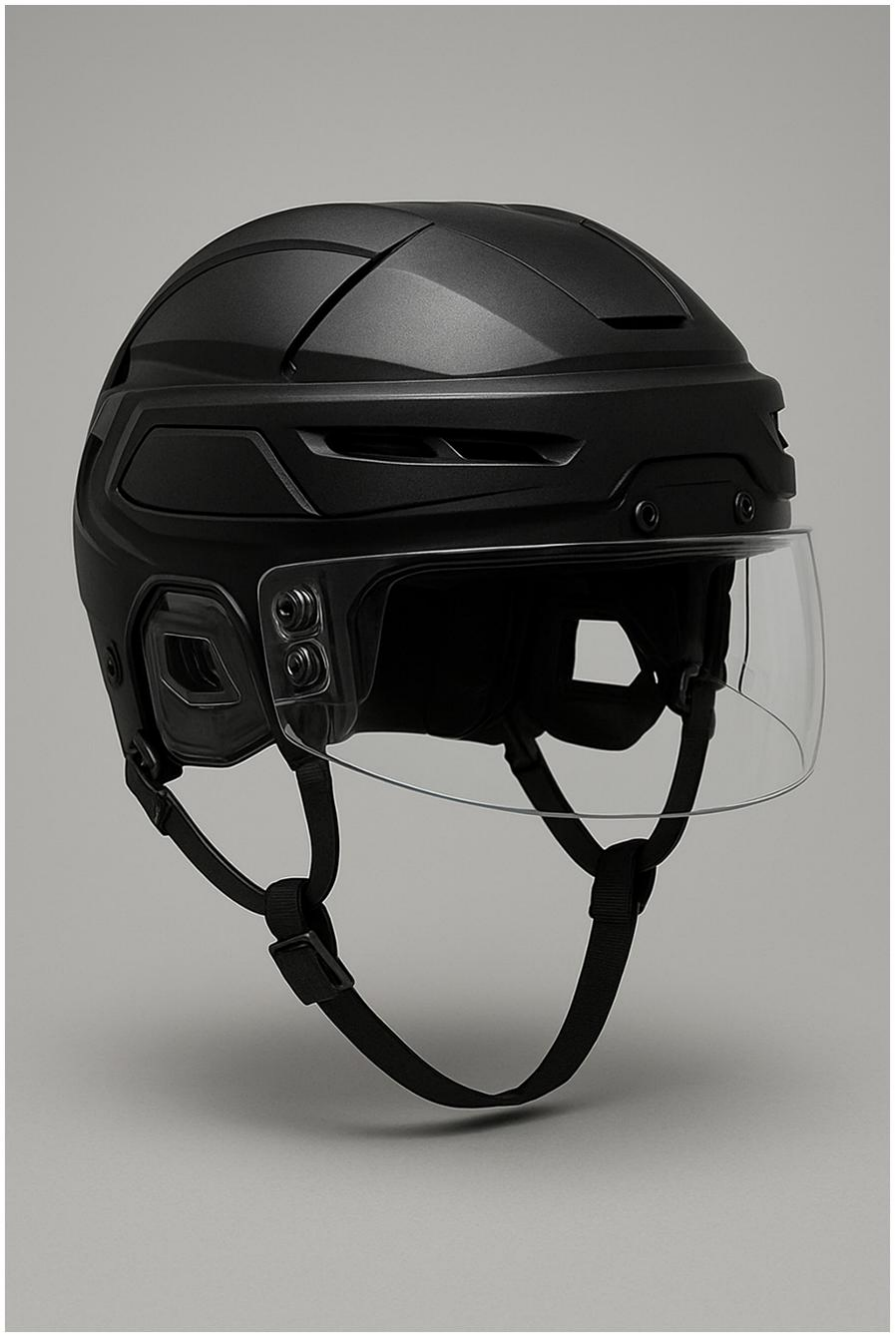
Ice hockey helmet with a shield visor.

An ice hockey game is played on a white ice surface called the rink, surrounded by smooth, white boards with a yellow kick plate at the base. Rink dimensions vary from 56 to 61 m in length and 26 to 30 m in width, with rounded corners (radius of 7 to 8.5 m). Protective glass, ranging from 80 to 200 cm in height, is installed above the boards, especially at the ends and sides of the rink, to protect players. All doors swing away from the ice, and only designated markings and advertising are allowed according to official regulations. Helmets and eye protector shields (Figure 1) are mandatory in ice hockey to safeguard players from head injuries, including concussions and skull fractures, as well as to protect the eyes from pucks, sticks, and other potential hazards that can cause serious injury during high-speed play (4).

Studies report higher concussion rates in North American leagues than in European ones, reflecting both contextual and methodological influences (5). The more physical North American playing style (6), greater player aggression (7), and a smaller rink size (8) may contribute to these differences. Yet diagnostic inconsistencies, such as variations in how concussions are defined and recorded, complicate cross-regional comparisons and may obscure actual incidence patterns (5).

It is estimated that 20%–25% of players in youth hockey sustain at least one concussion each season (9, 10). Similarly, concussions are common in the National Hockey League, occurring at a rate of 6 per 100 games, with concussions accounting for 14% to 30% of all hockey-related head injuries (11). Based on a systematic review, the incidence of concussions in the NHL ranged from 0.54 to 1.18 per 1,000 athlete-exposures between the 2000–2001 and 2022–2023 seasons (12). Based on this premise, the prevalence of concussions in hockey remains notably high across various levels, although inconsistencies in diagnosis hinder accurate comparisons.

Youth players face higher concussion risks than older players, as increased risk during early adolescence is due to the disparity in size, strength, and speed among boys at this age (13). Additionally, the body checking at a younger age can lead to injuries caused by poor checking techniques and a lack of awareness on the ice, resulting in more collisions (13). Games present significantly greater risks than practices, with illegal contact and bodychecking being significant factors (14). Evidence suggests that stricter rule enforcement and flexible rink designs can help reduce these risks (15). Biomechanical studies further reveal that long-duration, high-acceleration impacts are key injury mechanisms, highlighting the need for better helmet standards (16). Emerging smart helmets that incorporate sensor technologies and adaptive materials are promising advancements, building on innovations from other sports like cycling and equestrian activities (17–19).

## The limitations of current helmets

Recent research into helmet safety (Table 1) highlights several key advancements and challenges. Meehan et al. (20) developed realistic concussion-simulating tests for ice hockey helmets, urging improvements in testing standards. Champoux et al. (21) applied this protocol to three helmet models, finding minimal performance differences, suggesting a need for more innovative designs. Kolstad et al. (22) found that mouthguards significantly reduce the risk of concussion, while helmet age had no effect, supporting the mandatory use of mouthguards in youth hockey. Haid et al. (23) demonstrated that mechanical metamaterials offer design flexibility and superior energy management. Research by Tse and Holder (18) and Abderezaei et al. (17) has shown that airbag systems and rotational-damping technologies enhance protection, suggesting a potential application in hockey helmet integration. Giusti Gestri (19) and Goutnik et al. (24) emphasized the role of sensor-based feedback and cross-disciplinary approaches in advancing helmet safety and standards. Standard ice hockey helmets are primarily designed to protect against skull fractures and superficial head injuries. However, they provide limited protection against concussions (20, 22, 23, 25). They fall short in addressing concussions, which are caused not by direct impact to the skull but by the sudden acceleration or deceleration of the brain inside the cranium (26). Modern helmet designs use foam padding to absorb energy. However, this static approach falls short in reducing rotational forces, which are recognized as playing a central role in concussion mechanisms (27).

**Table 1:** Summary of helmet safety studies.

Study	Year	Helmet type	Testing method	Key metrics	Performance summary	Recommendations
Abderezaei et al. (17)	2021	Helmets with different technologies	Bicycle helmet drop tests	Peak rotational acceleration (PRA), peak linear acceleration (PLA), generalized acceleration model for brain injury threshold (GAMPIT)	Rotation-damping and collapsible technologies improved protection	Incorporate rotation management features in future designs
Tse and Holder (18)	2021	Airbag-integrated bicycle helmet	Simulated dynamic impacts	Head kinematics, brain injury metrics	Outperformed standard helmets, even when airbags did not deploy	Explore airbag systems for hockey helmet applications
Giusti Gestri (19)	2023	Jockey safety vests and sensors	Qualitative, user-informed design	Potential for real-time injury data	Sensor integration may enhance protective equipment design	Apply sensor-based feedback/diagnostics in helmets
Meehan et al. (20)	2022	Ice hockey helmets	Ice drop, angled board drop, pneumatic ram	Max Principal Strain (MPS)	Replicated realistic concussion-causing impacts	Use findings to improve helmet test standards
Champoux et al. (21)	2024	Ice hockey helmet designs	Meehan's protocol	Linear and rotational acceleration, Max Principal Strain (MPS)	Only small performance differences across helmet models	Designs may be too similar; greater innovation is needed
Kolstad et al. (22)	2023	Mouthguard, helmet age	Real-world cohort and injury surveillance	Concussion rate and odds ratio	Mouthguards significantly reduce concussion risk; helmet age is not a risk factor	Making mouthguards mandatory in youth hockey
Haid et al. (23)	2023	Mechanical metamaterial-based helmets	Structural and topology optimization	Linear and rotational acceleration	Provided design flexibility and energy management	Encourage the adoption of metamaterials in sports helmets
Goutnik et al. (24)	2022	Sports and military helmets	Literature review	Protection mechanisms, test standards	Highlighted helmet design gaps and injury mechanisms	Support cross-discipline innovation and updated standards
McGillivray et al. (25)	2022	Comparison of materials in helmet liners	Static and dynamic lab tests	Energy absorption, head acceleration	Thermoplastic polyurethane (TBU) outperformed vinyl-nitrile and was comparable/better than expanded polypropylene (EPP)	Recommend TPU/EPP combination for enhanced protection

## Future technologies in concussion prevention

Ice hockey helmets still perform poorly compared to headgear used in other sports (28). The most innovative head protection has emerged from sports such as horse riding, cycling (18), and football (29). Devices equipped with accelerometers and gyroscopes can measure the magnitude and direction of impacts in real-time, providing valuable data to coaches, medical staff, and researchers (30). For instance, an Australian qualitative study recommended further research on wearable sensors to enhance the protection offered by jockeys' safety vests (19). Research in equestrian sports has demonstrated the potential of embedding wearable sensors into protective gear, providing real-time injury feedback and diagnostic capabilities that could be applied to helmets (19). These systems are already being tested in American football, where early detection has led to quicker removal and evaluation of potentially concussed players (30). These findings suggest that adopting sensor technologies could substantially advance concussion prevention in ice hockey.

A more revolutionary concept is the use of smart technologies such as airbag systems in helmets. While this may sound far-fetched, prototypes already exist in other sectors. For example, airbag and rotation-damping helmets have been shown to significantly reduce the risk of brain injury in cycling (17, 18). An air-filled bicycle helmet for cyclists features an expandable airbag that protects the head, and an additional airbag designed to prevent neck injuries, both of which inflate within milliseconds upon activation (18). The principle is sound: rather than passively absorbing force, these devices dynamically respond to impacts, reducing both linear and rotational acceleration. Beyond hockey, evidence from cycling highlights the protective benefits of rotational-damping technologies, which substantially reduce brain strain in oblique impacts (17).

While airbag-integrated helmets have shown promise in other sports (17, 18), adapting such systems to ice hockey helmets presents engineering challenges. Unlike cycling, hockey involves frequent, multidirectional collisions in confined spaces, where both linear and rotational accelerations contribute to the risk of concussion (20, 21). Airbag systems, which rely on rapid inflation and increased helmet volume for protection, may be impractical in this context due to fit, weight, and mobility constraints, as well as their limited ability to manage rotational accelerations (24). Moreover, hockey helmets must endure repeated impacts within a single game, a demand that single-deployment airbag designs cannot meet. Despite challenges (e.g., deployment speed, false positives, and comfort issues), advances in sensors, batteries, and artificial intelligence suggest that airbag helmets could become commercially viable within a few years.

To accelerate progress in ice hockey, researchers, engineers, and sports scientists must coordinate efforts to translate existing technologies into rink-ready applications. The ethical case for smarter head protection is particularly strong in youth hockey, where developing brains are more susceptible to long-term damage (31). While professional athletes, such as those in the NHL, may accept a certain level of risk for their careers, younger athletes deserve better safeguards. Relying solely on rule changes or better coaching is not enough. Without a technological leap, we are simply accepting a baseline of injury that science may already have the tools to lower. Some scepticism exists around integrating complex tech into an already gear-heavy sport. Concerns include cost, weight, reliability, and whether players would accept such changes. But this resistance has historical precedent: when Jacques Plante first wore a goalie mask in 1959, the idea was ridiculed. Today, no goaltender would play without one. Similarly, carbon fibre sticks, once dismissed as fragile, are now ubiquitous. The trajectory of innovation shows that once safety technologies prove effective and scalable, acceptance follows.

## Conclusion

The problem of concussion in ice hockey is not new, but the tools to address it are evolving rapidly. From sensor-embedded helmets to the potential of airbag systems, technology presents an unprecedented opportunity to transform the landscape of injuries. Integrating innovative materials, real-time data, and dynamic response systems could significantly reduce concussions in both professional and amateur hockey. As with many disruptive innovations, it is not a question of *if* these tools will be adopted, but *when*. The game can evolve without losing its identity. The only real obstacle is the common willingness to embrace the future.
